# Supplementary material for: Accelerated multi‐shell diffusion MRI with Gaussian process estimated reconstruction of multi‐band imaging
Source: Magn Reson Med. 2025 Apr 6;94(2):694–712. doi: 10.1002/mrm.30518 (PMC12137782; doi:10.1002/mrm.30518)
Supplement: Supplementary file 1 — Table S1. In vivo acquisition protocols. Table S2. Weighted‐DICE values between the tracts obtained from singleband reference and the tracts from different reconstruction methods. Figure S1. Difference maps between reference and reconstruction results of the 36‐direction 1.5 mm isotropic resolution in vivo data shown in Figure 5. Compared to SENSE and ss‐DAGER, ms‐DAGER shows least error. Figure S2. Reconstruction results of the 1.5 mm isotropic resolution in vivo data from subject 1. Single band reference images with 2 average, SENSE and ms‐DAGER results are shown b = 1000s/mm2 (‘b1k’) and b = 2000s/mm2 (‘b2k’) images are both shown. Difference maps between reconstruction results and reference are shown. ms‐DAGER consistently improve image quality compared to SENSE for both subjects. Figure S3. Reconstruction results of the 1.5 mm isotropic resolution in vivo data from subject 4. Single band reference images with 2 average, SENSE and ms‐DAGER results are shown b = 1000s/mm2 (‘b1k’) and b = 2000s/mm2 (‘b2k’) images are both shown. Difference maps between reconstruction results and reference are shown. ms‐DAGER consistently improve image quality compared to SENSE for both subjects. Figure S4. Coronal and sagittal slices of 1.5 mm isotropic resolution in vivo data. Single band reference images with 1 average, SMS = 2 SENSE, SMS = 4 SENSE and SMS = 4 ms‐DAGER results are compared. b = 1000s/mm2 (‘b1k’) and b = 2000s/mm2 (‘b2k’) images are both shown. ms‐DAGER provides improved SNR compared to SMS = 4 and SMS = 2 SENSE, with comparable data quality to the reference images. Note image contrasts are slightly different between methods due to different TR used. Figure S5. (A) Error maps between DKI fitting results from reference and reconstruction results for the 1.5 mm isotropic resolution in vivo data shown in Figure 8. Median absolute error values are also shown for Mean kurtosis (MK), axial kurtosis(AK) and radial kurtosis(RK). (B) Error maps between NODDI fitting resul [file MRM-94-694-s001.docx]

**Supporting information:**


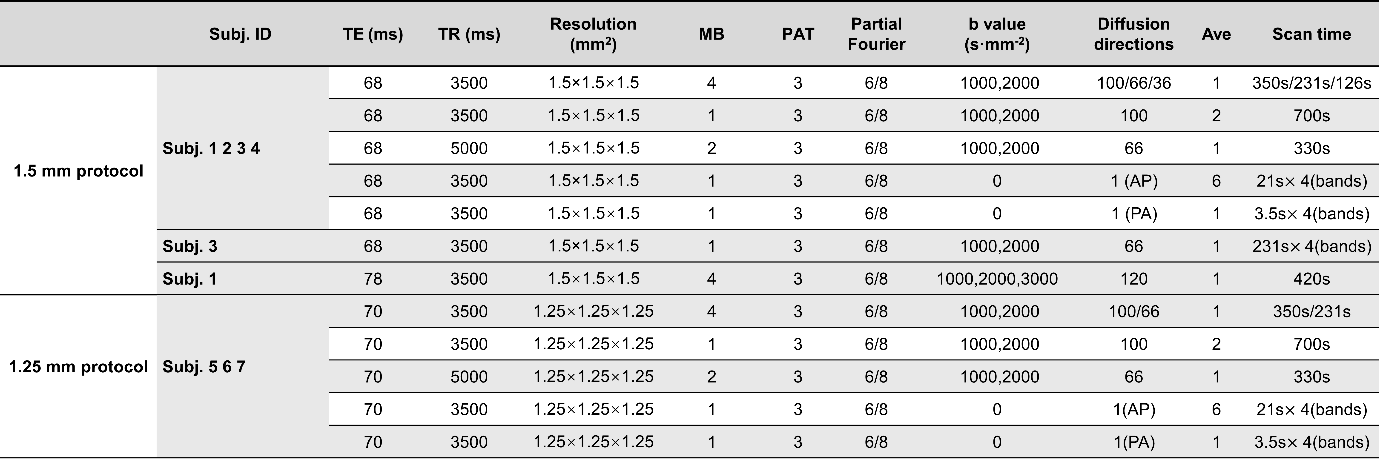


Table.S1 In vivo acquisition protocols.

| Tracts | SENSE | ms-DAGER |
| --- | --- | --- |
| atr | 0.8200 | 0.9526 |
| str | 0.8672 | 0.9629 |
| ptr | 0.7159 | 0.9345 |
| fmi/fma | 0.7613 | 0.8995 |
| cst | 0.9285 | 0.9779 |
| ar | 0.3820 | 0.8093 |
| ml | 0.7181 | 0.7253 |
| mcp | 0 | 0.3515 |
| unc | 0.5765 | 0.7561 |
| cgh | 0.3121 | 0.7368 |
| cgc | 0.9711 | 0.9714 |
| ifo | 0.4733 | 0.8548 |
| ilf | 0.3778 | 0.7964 |
| slf | 0.8984 | 0.9550 |

Table.S2 Weighted-DICE values between the tracts obtained from singleband reference and the tracts from different reconstruction methods.

Fig.S1 Difference maps between reference and reconstruction results of the 36-direction 1.5 mm isotropic resolution in vivo data shown in Fig.5. Compared to SENSE and ss-DAGER, ms-DAGER shows least error.

Fig.S2 Reconstruction results of the 1.5 mm isotropic resolution in vivo data from subject 1. Single band reference images with 2 average, SENSE and ms-DAGER results are shown b=1000s/mm^2^ (‘b1k’) and b=2000s/mm^2^ (‘b2k’) images are both shown. Difference maps between reconstruction results and reference are shown. ms-DAGER consistently improve image quality compared to SENSE for both subjects.

Fig.S3 Reconstruction results of the 1.5 mm isotropic resolution in vivo data from subject 4. Single band reference images with 2 average, SENSE and ms-DAGER results are shown b=1000s/mm^2^ (‘b1k’) and b=2000s/mm^2^ (‘b2k’) images are both shown. Difference maps between reconstruction results and reference are shown.ms-DAGER consistently improve image quality compared to SENSE for both subjects.


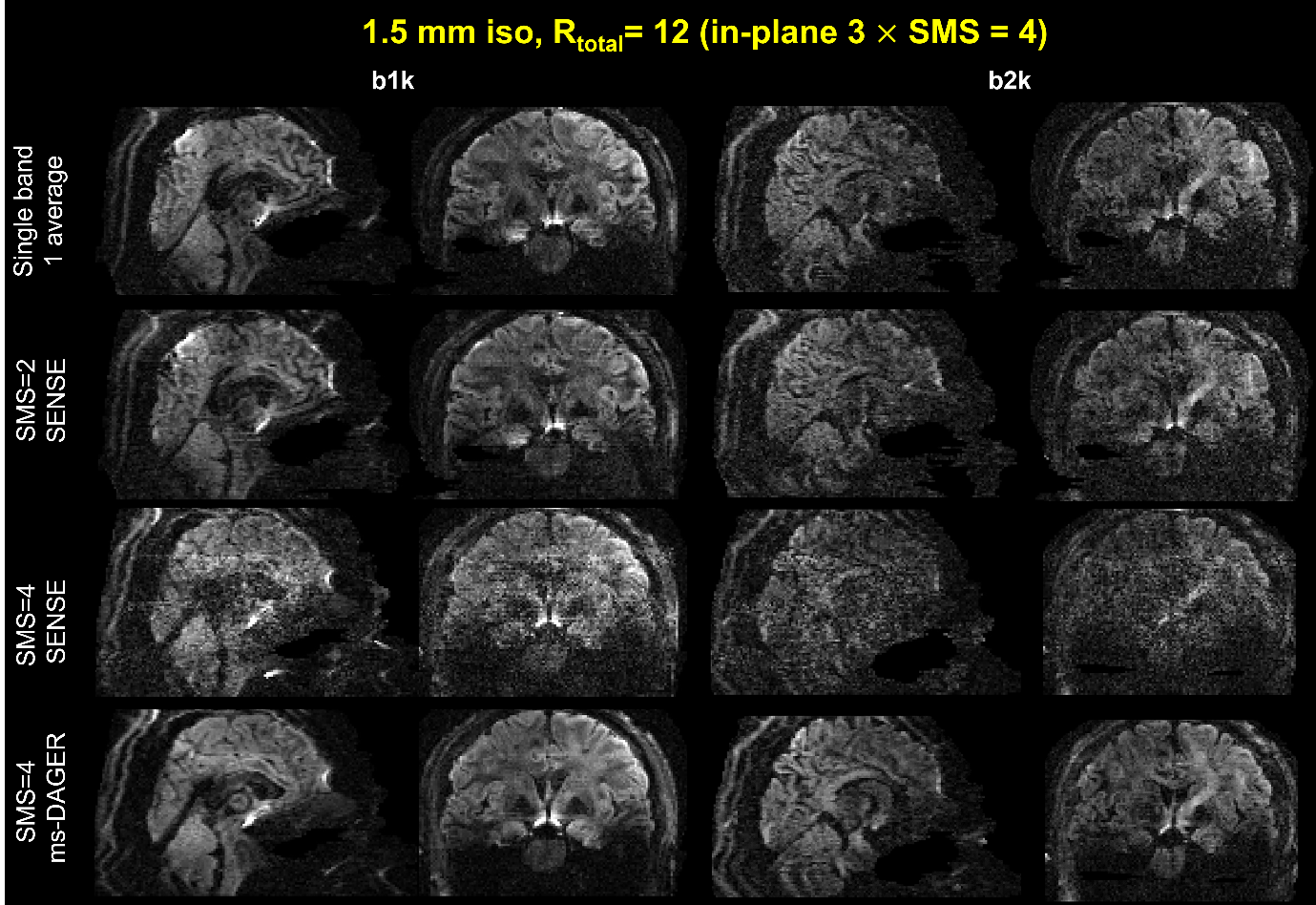


Fig.S4 Coronal and sagittal slices of 1.5 mm isotropic resolution in vivo data. Single band reference images with 1 average, SMS=2 SENSE, SMS=4 SENSE and SMS=4 ms-DAGER results are compared. b=1000s/mm^2^ (‘b1k’) and b=2000s/mm^2^ (‘b2k’) images are both shown. ms-DAGER provides improved SNR compared to SMS=4 and SMS=2 SENSE, with comparable data quality to the reference images. Note image contrasts are slightly different between methods due to different TR used.

Fig.S5 a)Error maps between DKI fitting results from reference and reconstruction results for the 1.5 mm isotropic resolution in vivo data shown in Fig.8. Median absolute error values are also shown for Mean kurtosis (MK), axial kurtosis(AK) and radial kurtosis(RK). b)Error maps between NODDI fitting results from reference and reconstruction results for the 1.5 mm isotropic resolution in vivo data shown in Fig.9. Median absolute error values are also shown for CSF volume fraction(fiso), intra-cellular volume fraction(fintra) and orientation dispersion index (ODI).


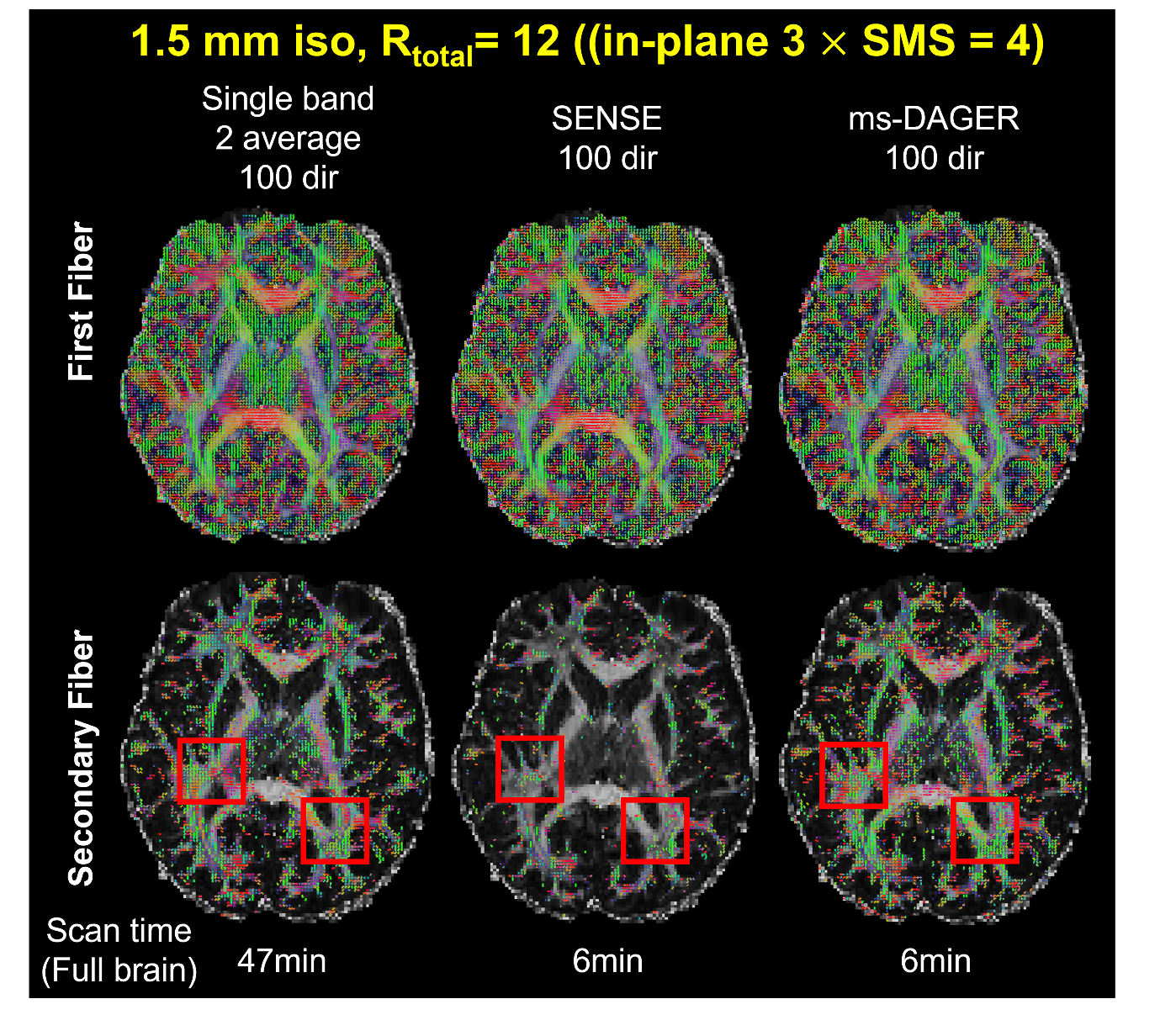


Fig.S6 Ball and stick fitting results for the 1.5 mm isotropic resolution in vivo data. The first fiber and second fiber populations calculated from single band reference images with 2 average, SENSE and ms-DAGER results are shown. ms-DAGER recovers a large number of second fiber population, consistent with the single-band reference, while SENSE results fail to capture many second fibers.


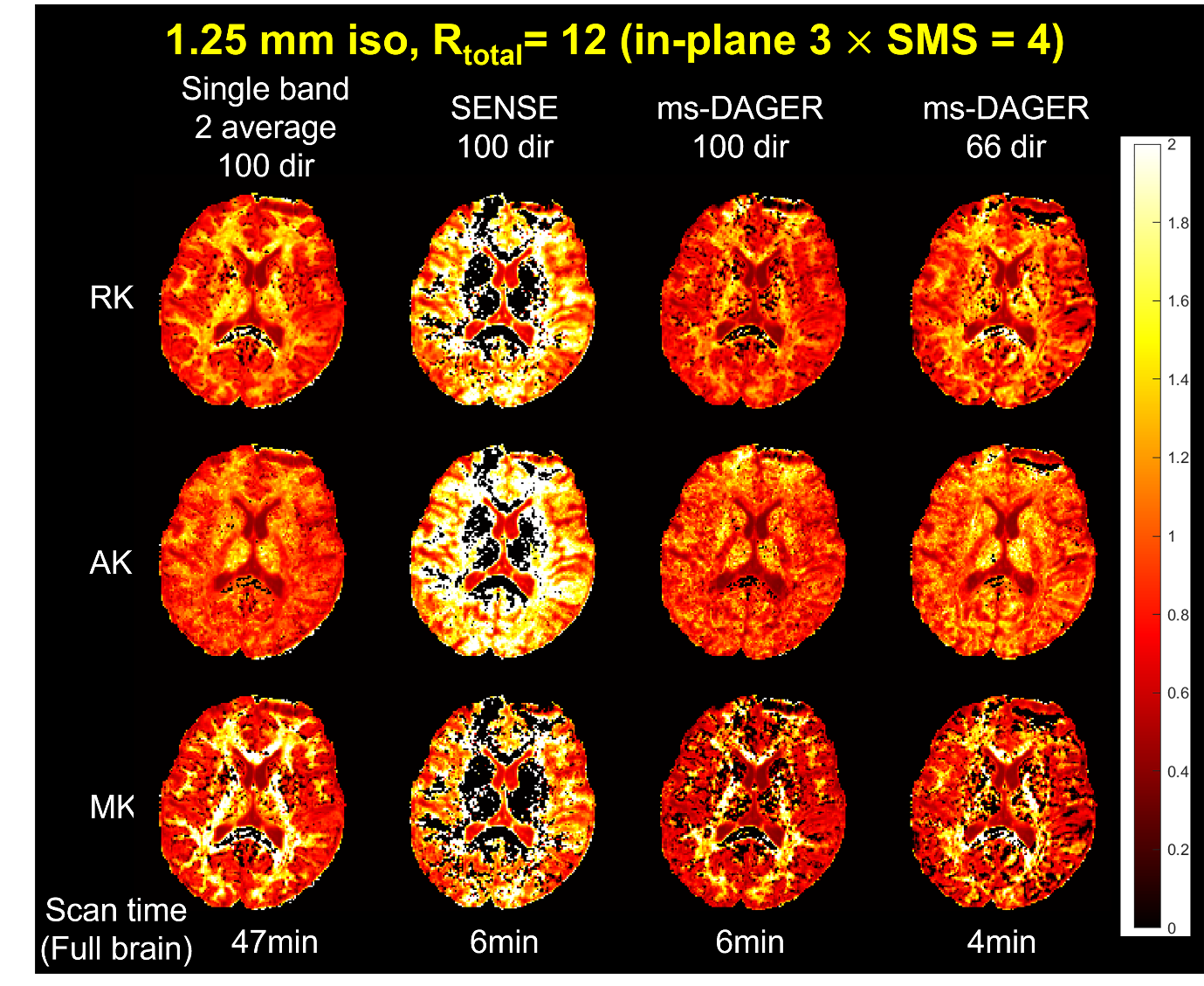


Fig.S7 DKI fitting results for the 1.25 mm isotropic resolution in vivo data. Mean kurtosis (MK), Axial kurtosis(AK) and Radial kurtosis(RK) maps calculated from Single band reference images with 2 average, SENSE and ms-DAGER results are shown. Compared to SENSE, ms-DAGER produces more consistent results with reference using much shorter scan time.


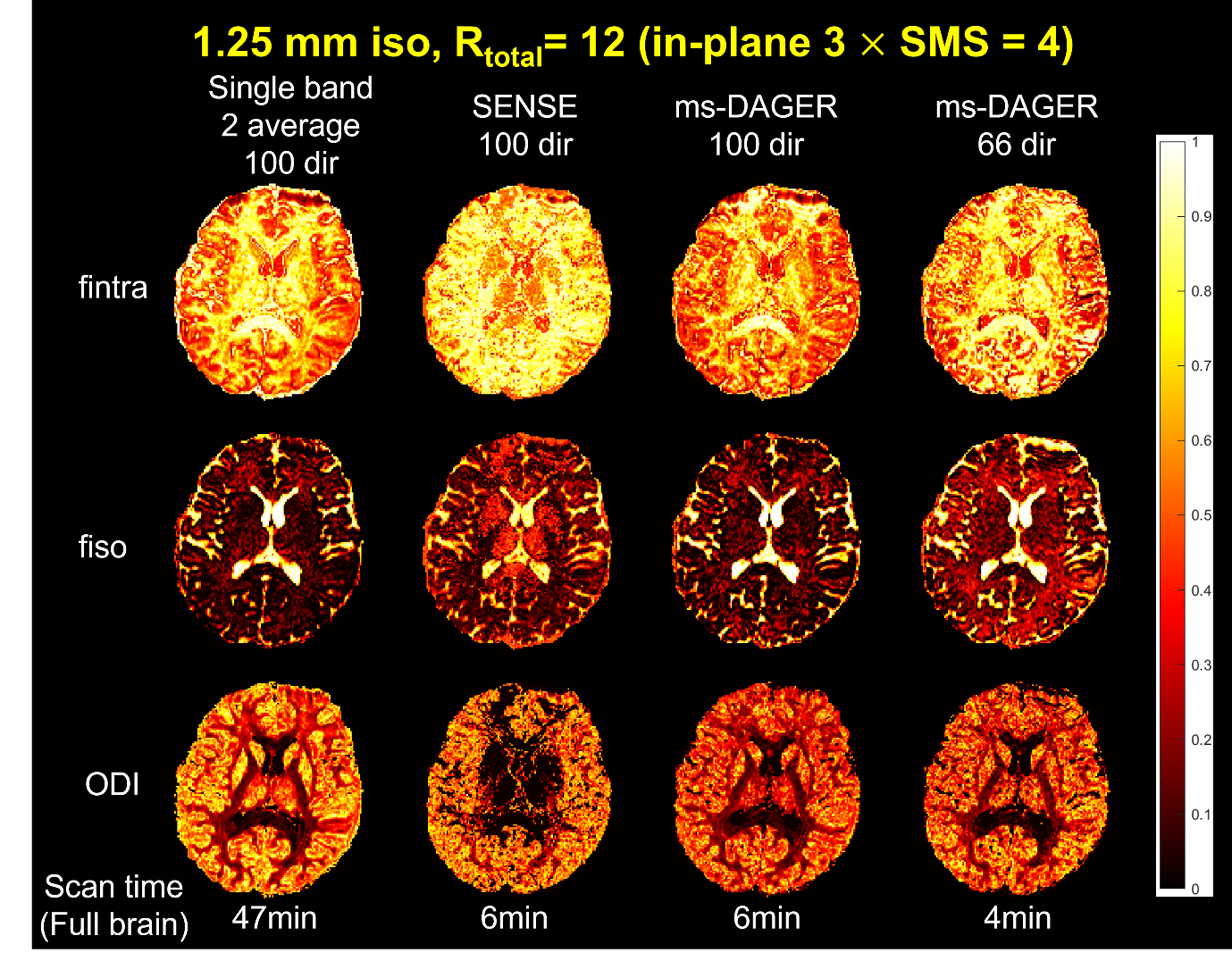


Fig.S8 NODDI fitting results for the 1.25 mm isotropic resolution in vivo data. CSF volume fraction(fiso), intra-cellular volume fraction(fintra) and orientation dispersion index (ODI) from NODDI calculated from Single band reference images with 2 average, SENSE and ms-DAGER results are shown. Though bias exists in ODI maps, ms-DAGER can produce improved fitting results compared to SENSE results which are significantly corrupted by noise.

Fig.S9 a)Error maps between NODDI fitting results from reference and reconstruction results for the 1.25 mm isotropic resolution in vivo data shown in Fig.S8. Median absolute error values are also shown for CSF volume fraction(fiso), intra-cellular volume fraction(fintra) and orientation dispersion index (ODI). b)Error maps between DKI fitting results from reference and reconstruction results for the 1.25 mm isotropic resolution in vivo data shown in Fig.S7. Median absolute error values are also shown for Mean kurtosis (MK), axial kurtosis(AK) and radial kurtosis(RK).


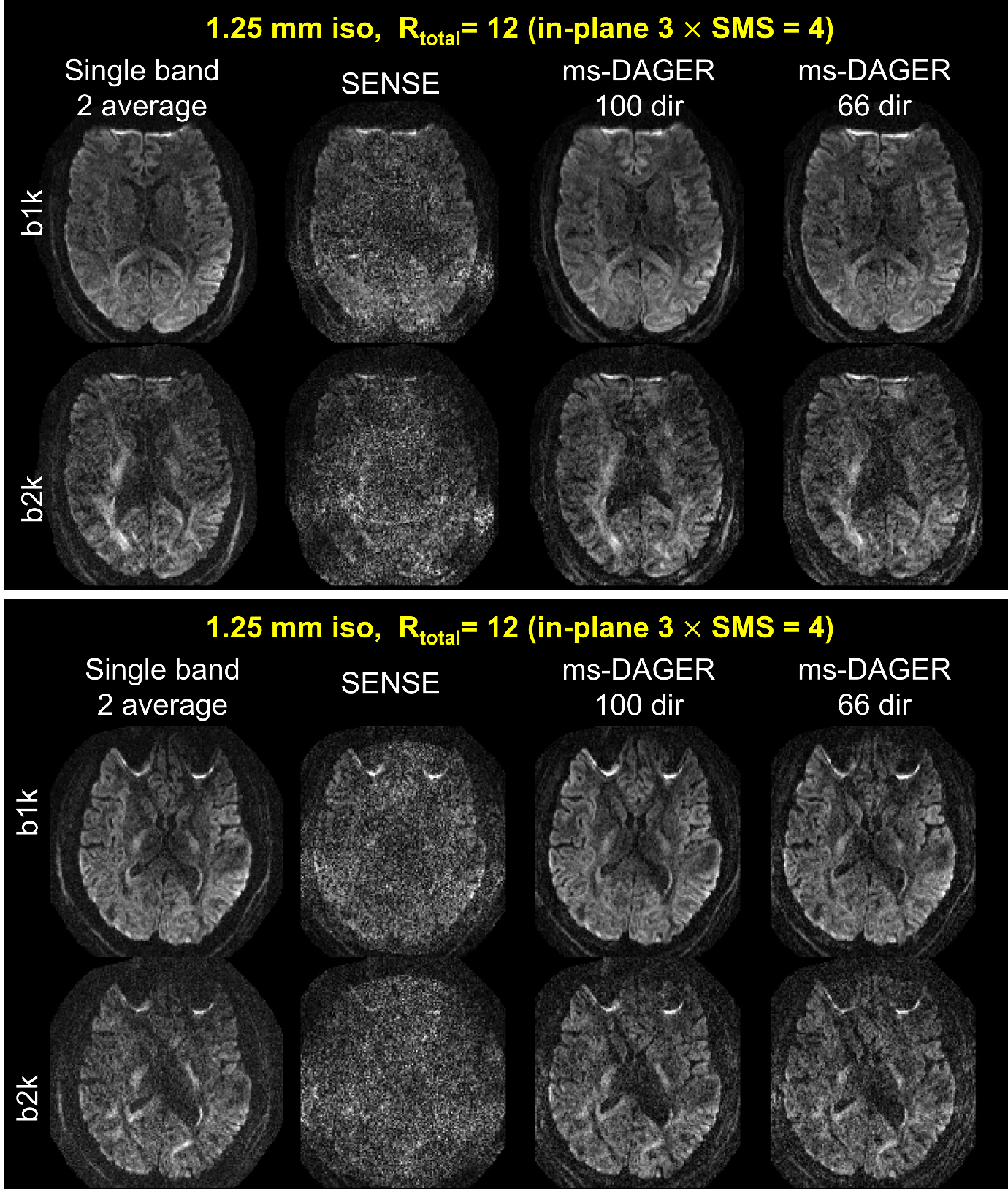


Fig.S10 Reconstruction results for the 1.25 mm isotropic resolution in vivo data from two other subjects. Single band reference images, SENSE and ms-DAGER results are shown. b=1000s/mm^2^ (‘b1k’) and b=2000s/mm^2^ (‘b2k’) images are both shown. ms-DAGER can consistently improve image quality.


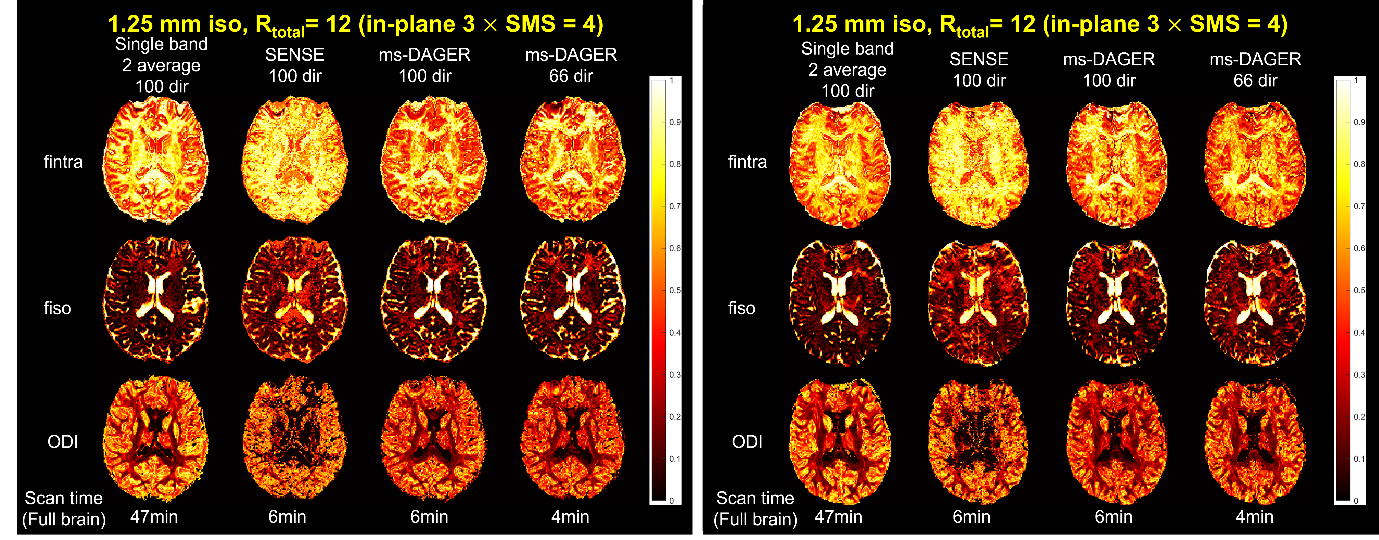


Fig.S11 NODDI fitting results for the 1.25 mm isotropic resolution in vivo data for other subjects. CSF volume fraction(fiso), intra-cellular volume fraction(fintra) and Orientation dispersion index (ODI) from NODDI calculated from Single band reference images with 2 average, SENSE and ms-DAGER are shown are shown. Compared to SENSE, ms-DAGER consistently improve the fitting accuracy, showing high reproducibility across subjects

Fig.S12 Difference maps between reference and reconstruction results for 1.25 mm isotropic resolution in vivo data from two other subjects shown in Fig.S10. Note that ms-DAGER method largely reduce the error compared to SENSE.

Fig.S13 Error maps between NODDI fitting results from reference and reconstruction results for the other 1.25 mm isotropic resolution in vivo data shown in Fig.S11. Median absolute error values are also shown for CSF volume fraction(fiso), intra-cellular volume fraction(fintra) and orientation dispersion index (ODI).

Fig.S14 (a) ODI maps from NODDI fitting of simulation data with different noise levels (SNR15 and SNR 7.5). The noisy datasets and the noise-free reference were fitted with NODDI. (b) Box plots showing ODI distribution within white matter region and gray matter region, respectively, for different noise levels.


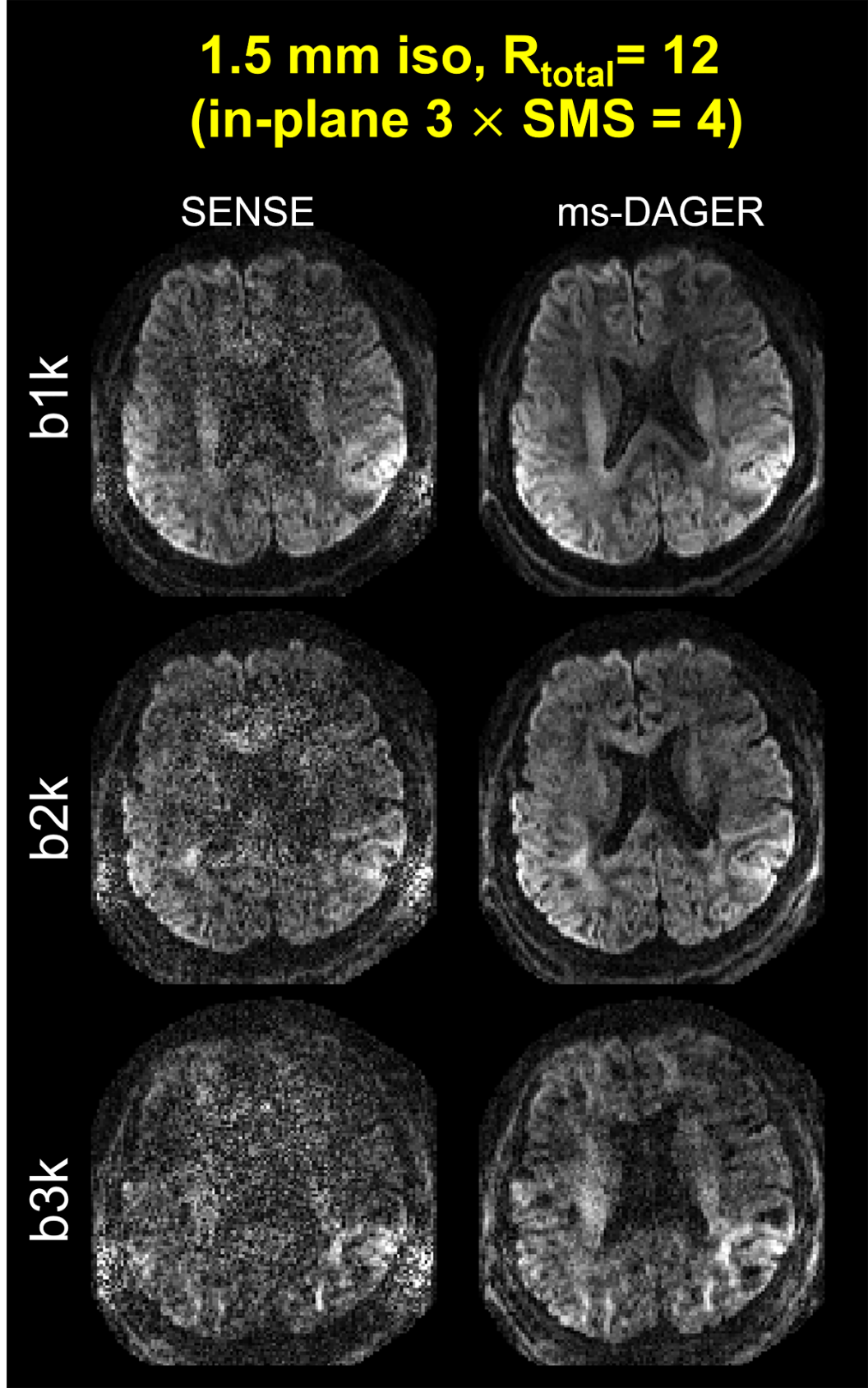


Fig.S15 Reconstruction results for the 1.5 mm isotropic resolution 3-shell in vivo data. SENSE and ms-DAGER results are shown. b=1000s/mm^2^ (‘b1k’) , b=2000s/mm^2^ (‘b2k’) and b=3000s/mm^2^ (‘b3k’)images are both shown. The image quality of higher b value shell can be significantly improved compared to SENSE with the help of cross-shell information sharing.
